# Student experience and digital storytelling: Integrating the authentic interaction of students work, life, play and learning into the co-design of university teaching practices

**DOI:** 10.1007/s10639-022-11566-8

**Published:** 2023-04-03

**Authors:** Peter Bryant

**Affiliations:** University of Sydney Business School, Sydney, Australia

**Keywords:** Student experience, Digital storytelling, Digital media, Pedagogical change, Co-design of teaching

## Abstract

Designing strategic pedagogical change through the lens of a student experience that is *yet to be experienced* offers a critical frame for embedding the impacts of transition, uncertainty, belonging and the complexity of the student journey into the co-design of teaching and learning. A digital storytelling approach extends the notion of the student experience beyond the singular and metricised descriptions common in online student satisfaction survey instruments into a rhizomatic, resonant living community that resides in the intersecting spaces of work, life, play and learning. This paper describes an ethnographic-like model of collecting and evaluating the student experience through a semi-structured digital storytelling methodology that supports both co-design and cogenerative dialogue as a form of curriculum enhancement. The paper outlines how the Student Experience Digital Storytelling model was iteratively designed, deployed, and then evaluated through participatory action research-informed case studies at the University of Sydney Business School (Australia) and the London School of Economics and Political Science (United Kingdom) that embedded the student experience into the co-design of curriculum and assessment interventions.

## Introduction

For such a widely used descriptor, there are significant inconsistencies in how the term *student experience* is defined and applied in higher education practice and literature. It has been used to represent the students feelings, expectations, engagement with pedagogy and assessment and how they interact socially and with the university (Cano et al., 2021). Evolving from an understanding of the satisfaction the student cohort has with teaching, the conceptual and message framing of the student experience has been expanded to include the student experience of student administration and ancillary services like car parking and food service (Douglas et al., 2008), as well the importance placed by students on social connections, transition and a sense of belonging during and after their higher education experience (De Sisto et al., 2022). The definition has been further fractured through the articulation of the student experience into the evaluations of first year transition pedagogies, student wellbeing strategies (Hayes & Jandrić, 2021) and institutional branding and marketing plans (Chen, 2022).

Whilst there remains contention between definitional frames, the use of data that measures the student experience remains a critical antecedent to enhancing the educational effectiveness of curriculum and improving the satisfaction and engagement students have with their education (Baird & Gordon, 2009; Booth, 2013). Students are the experts in interrogating and sharing their own experiences of learning (countering the fact that most teachers are not experts in being a modern student) (Gibbs & Wood, 2021). The incorporation of the authentic student experience enables students to act as collaborators and partners in the teaching and learning design process (Healey et al., 2014).

Student satisfaction is one of the most (over) used measures of the student experience designed in part to find the simplest, most comparable metrics of a student’s educational activities and their utility with the outcomes of that experience (Dean & Gibbs, 2015). Online student satisfaction surveys are one of the most common student experience data collection instruments, deployed at or near the completion of a unit of study or at various stages throughout a qualification by the awarding institution (Kane et al., 2008; Yorke, 2013) or by national accrediting or regulatory bodies (such as the Quality Indicators of Learning and Teaching in Australia and the National Student Survey in the United Kingdom). Their primary purpose is to determine the satisfaction of students with their educational experience, metricised against a limited number of pre-determined criteria and aggregated at a cohort, degree, or institutional level (Shah et al., 2021). They can reduce the measurement of student satisfaction to relatively narrow intervals on Likert scales and anonymous qualitative comment, reported as abstract from the students themselves and recorded at a single, historical point in time.

Online student satisfaction surveys represent the student experience as dissonance or reinforcement of expectations over experience, where an experience that meets or exceeds expectations is considered satisfactory and one that does not meet expectations is ranked relatively poorly (Abizada & Mirzaliyeva, 2021). After the student has engaged in learning there is little a teacher or their institution can do to change this dissonance or reinforcement, with the survey acting as a summative tool providing feedback on what has been experienced, as opposed to informing curriculum design in real-time whilst is being experienced (or even yet to be experienced for a future learning opportunity) (McDonald et al., 2021).

The student experience metrics derived from the student satisfaction data can promote and reward reactive and reductive decision making by academics, institutional management or the government setting the policy agenda for higher education (Klemenčič & Chirikov, 2015). The efficacy of online student satisfaction surveys for informing the enhancement of curriculum is impacted by low response rates (Ogden & Ogden, 2018), gender and other biases (Papadopoulou et al., 2019) or associations with neo-liberal agendas (Ball, 2016). The legitimisation that student satisfaction data gives to the notion of student as a customer of the institution or their role as rational technical actors engaging in university activity for the utilitarian outcome of employability, directly influences how the dialogue generated by the data can be represented with a consumerist moral authority (Grebennikov & Shah, 2013; Sabri, 2011).

The absolutism of a consumerist perspective applies an instrumental lens to how students rate their university experience, valuing high grades or the volume of assessment feedback over more abstract notions of satisfaction with an overall experience (Wong & Chiu, 2019). Online student satisfaction surveys can conflate notions of happiness with satisfaction and achievement of outcomes, especially as a marketised higher education system privileges consumerist language in the description of experience (Elwick & Cannizzaro, 2017). Dean and Gibbs (2015) argue that ‘identifying profound happiness as a goal for student development, rather than just satisfying their needs (the two are not exclusive), helps to focus the edifying mission of the university and so keep its distinction’ (p. 16). Such conflation can also reinforce the idea of student satisfaction as either a type of settling up (as in contentment with the transactional cost versus the outcomes of their education, such as desired employment) or settling down (contentment with the meeting of their needs or expectations of the experience itself) (Skea, 2017), failing to record and recognise the value of unsettlement with the less explicit senses of being and space experienced by students. In the Heideggerian sense, unsettlement is not a negative state, but emerges from feeling ‘unhomely’ with your own being. It is this angst at the ontological state of not knowing that can be educative within their learning experiences and thereby positive (Withy, 2015). The ideographic challenge for those engaging in curriculum design is to identify methodologies that embed the subjective, uncertain, and uncanny experiences of student learning and belonging into both the design of curriculum and how students can have agency over how they are recognised, shared, and represented in authentic and co-designed ways.

## Case studies

### LSE2020 (2016–2018)

The London School of Economics and Political Science (LSE) is one of the world’s leading social sciences universities. In 2016, it began a strategic process of pedagogical change in response to poor student experience results over three successive years in the United Kingdom National Student Survey (NSS), which pointed to a consistent decline in the student satisfaction with teaching based on the experiences of third-year undergraduate students. The visibility of the student journey, the variability of the experiences between courses and extra-curricular activities and lasting career-level impacts of graduating were not explicit in the measurement of attitudes and satisfaction from a self-selecting cohort of respondents. The ambition of these projects was to initiate a co-design process at a unit of study level through what Roth and Tobin (2005) refer to as a cogenerative dialogue, where teachers and students participate in conversations to enhance the effectiveness of teaching and learning (Gunckel & Moore, 2005). The summative and retrospective nature of the NSS data did not provide the immediacy of experience necessary for dialogue with most students surveyed having already finished their degree. For cogenerative dialogue to inform a co-designed curriculum, student assessments of teaching must pivot from being interrogated by the invisible hand of an assessor reporting on actions past towards understanding the efficacy of teaching through the lens of how teaching changes the students relations to the world and how the teaching encourages them to take action (Lave, 2009). At a unit of study level, cogenerative dialogue was especially relevant to understanding the disturbances that contributed to the overall, summative dissatisfaction with teaching, flattening the power structures to the point where feedback took a sense of immediacy often lacking in the university curriculum approval and governance processes (Milne et al., 2011).

### Case 2 – Work, Live, Play, Learn (WLPL) (2018–2022)

The University of Sydney Business School (USBS), Australia delivers programs for over 15,000 domestic and international students. The scale of the School (and the wider university) means students are distributed across a large and sprawling campus, in fractured groups and classes that do not afford many opportunities to connect and build strong links within a cohort. In 2018, the Business School developed a program of co-designed curriculum enhancement addressing the twin challenges of minimising the impacts of social isolation in a large and disparate student cohort and building connection and a sense of belonging through teaching and learning.

The University collects student satisfaction data on a unit-by-unit level, across ten 5-point Likert scaled questions, representing the experiences of students that have already completed their units of study. Before and during the COVID-19 pandemic, the complexities of the intersections and pressures of work, life and play and learning created impacts on teaching that were *being experienced* in situ*.* Reflecting on the iterative nature of the co-design process, where changes are made incrementally to adjust teaching, learning and assessment activities, the capacity for students to feed into the design of units they are yet to experience, along with the perspectives of those who have completed the units imbues the process with a true sense collaboration, research rigor and empathy (Dollinger et al., 2022). The intentions of the co-design process (called Connected Learning at Scale) was to create multiple opportunities and spaces where knowledge and skills could be shared by staff, students, the community, and the university, to engage in the making, leveraging and application of connections throughout their journey. Connected Learning at Scale privileged the social process of connecting and the social acts of making, doing, and sharing actions. The knowledge and skills acquired and applied through our connected learning process were designed to be lasting, transdisciplinary and trans-contextual. It is in this context of cogenerative dialogue that a richer source of student experience data that augmented the unit-level data gained from online student satisfaction surveys was critical.

## Methodology

This paper explores the evolution of a model of student experience data collection that emerged from strategic projects that were designed to enhance the design of curriculum at two leading research-intensive universities in the UK and Australia. Both of the projects were underpinned by the philosophy of co-design, which engages students as partners in the design of both curriculum and teaching and learning activity, informing the structure of delivery, the nature of assessment and iterative improvement of the design over time (Bryson, 2014; Wilson et al., 2021). A critical challenge when implementing co-designed curriculum is the limited effectiveness of student experience data that comes from student satisfaction surveys in representing the authenticity of the student experience and the capability to act as a frame for the students in the project to expand their own perceptions of teaching and learning into a wider, more representative view of the cohort. This challenge became the post-factum research question for this study, not determined whilst the projects were in action but arising from how the learning in these projects could be generalised and shared with other institutions through the development of a model (Schubert, 1996).

The projects explored in this study deployed an educational intervention in the field (the collection and analysis of student experience data to inform the co-design of curriculum). Participatory action research (PAR) was used to guide the methodological structure of the project and ensure research insights fed into the actions of participants (Kemmis, 2006; McTaggart, 1991). The use of PAR provided a structured approach to identifying specific problems impacting on curriculum design and through a collaborative and co-designed inquiry, generate solutions that were relevant not just to the context of the project and the institution, but beyond into other contexts (Baum et al., 2006; Stringer & Aragón, 2020). Each project produced critically reflective case studies in several different forms (reports, video artefacts and social media posts) sharing the reflections-on-action inside and outside the institution (Altrichter et al., 2002).

**Fig. 1:**
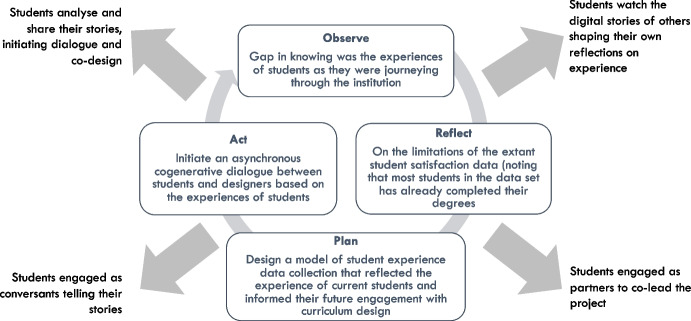
Expanded participatory action research cycle

Between 2014 and 2022, there were six projects (three at LSE and four at USBS) that were designed to collect and analyse the student experience to catalyse different cogenerative dialogues and inform projects of co-designed curricular change. Each project design included a PAR (Baum et al., 2006) to structure the methodological approach and then collectively reflect on and evaluate the effectiveness of the intervention (Fig. 1). As part of the reflective cycle of pedagogical action research posited by Norton (2014) the methodology of PAR informed how the action itself was designed, which in this case informed how the project was framed, how the data was collected and determined the evaluative framework that was used to determine impact. The principles of PAR are built on action as a way of empowering researchers and participants to reflect on practice and exert greater control over the lives. The projects were structured to ensure the students acted as both researcher and participant, affording them ownership over the data and its agency over how it was represented and used (McTaggart, 1991). Each reflective cycle of the project refined how the student experience data collection methodology was delivered through reflection-on-action and an evaluation of how the data supported cogenerative dialogue and co-design. This is what Roth and Tobin refer to as metaloguing where the practice of teaching and learning is theorised, and iteratively reflected back into policy, and then back into practice and theory through dialogue between students and teachers (Otrel-Cass & Fladkjær, 2017).

The collection of student experience data in these projects was designed as an ‘on-the-ground’ or ethnographic-like approach to understanding the lived authentic experience of our students, where the methodology is not pure ethnography but a blurred genre, drawing on multiple methodologies to elicit responses (Agar, 2010). The project teams comprised current students and recent graduates co-leading the data collection and analysis. The implemented and ‘real’ aspects of the PAR methodology were critical to ensuring the quality of the knowledge and practicality and usefulness of the outcomes, especially in relation to understanding the being and yet to be experienced modalities of learning. It also enabled the capability for students to reflect on the complexity of the experiences they were having at that moment, allowing for real-time feed-forward into the process of co-design (Phelps & Hase, 2002).

A collective critical reflection of the data and the methodological approach itself was undertaken by the project team at the completion of each PAR cycle. The model of analysis through reflection was built on constructivist grounded theory-informed approaches posited by Charmaz (2006), which recognises the critical importance of the positionality of the researcher and the influence of a posteriori knowledge in relation to the research question being explored. This approach also allowed for theory to be constructed through the process of conducting action research, drawing on both the perceptions of experience from the student themselves, from the ethnographic-like engagement of the research team and then the wider institution using the data.

## Introducing the student experience digital storytelling model (SEDS)

**Fig. 2:**
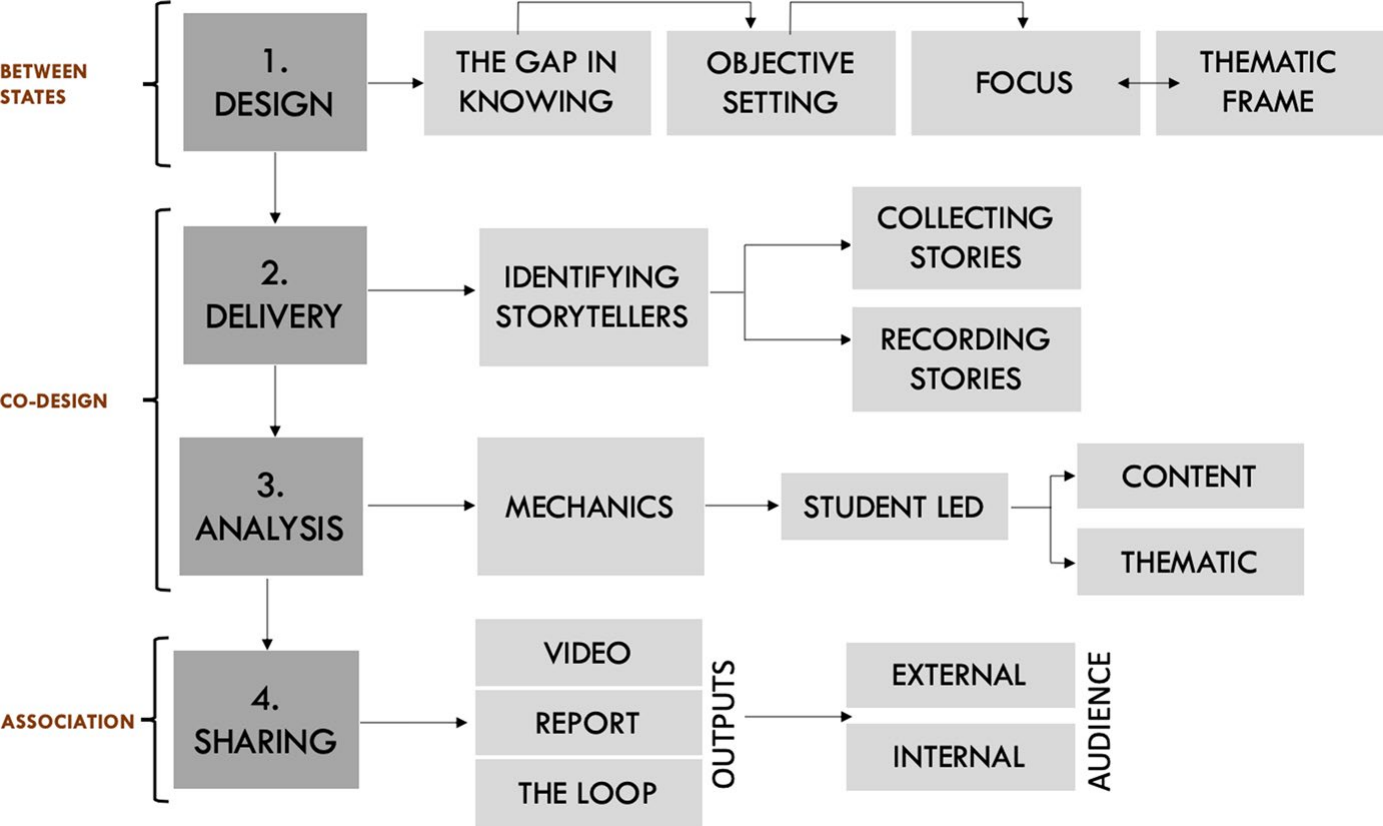
Student experience digital storytelling model

The student experience digital storytelling model (SEDS) emerged from the PAR projects as a methodological framework for the capturing, analysis, and dissemination of purposeful and holistic student experience data through digital stories, collected on video, analysed collectively, and shared using social media (Fig. 2). Aligned to the intention of cogenerative curricular design, the SEDS model locates the universities (and the student cohorts) understanding of the student experience within learning and life spaces that exist between an unknown or *yet to be experienced* future and a very present sense of now, rather than in the known or the *already experienced*. The liminal spaces of the *yet to be experienced* are hybridised, located within and between digital and physical presences and not exclusively bound by the experiences within the University. They are messily integrated into how students are living their lives. The connective tissue between these experiences is a fluid combination of ways of communicating and connecting, deployed, and used for both their affordances and their convenience. The use of the conceptual frame of the *yet to be experienced* represents the uncertainty of what is to come in their university experience, a future that is not designed by the summative experiences of others who have already completed that journey. This imbues the data with a deep realism and a sense of immersion and representation.

Digital storytelling became the narrative heart of the projects and the SEDS model. Digital storytelling practices using mobile or mobile-like media forms represents these current and the yet to be experienced in more exposing, engaging and integrated ways than online student satisfaction surveys can do because the stories and the people telling them reside and are created within these hybridised spaces (which are often virtual, deeply fragmented and beset with challenges of identify, visibility and voice) (Goggin & Hamilton, 2014). The use of digital stories imbues the data with non-visual cues, communicative tropes like voice tone, pitch, mood, gestures, movement and the capability to add visual prompts such as iPads, whiteboards and writing into the stories created through digital tools (Bearne & Wolstencroft, 2007). The visible and digital nature of the data in the SEDS model allows for the stories to shared, remixed, repurposed and used to create communities of affiliation and representation between current and future students (Rosen, 2022), which enhances the effectiveness of the cogenerative dialogue.

Through telling their stories on camera and sharing their experiences in conversations, students reflect actively on their learning and share the tensions and doubts that arise from the uncertainty they are feeling or experiencing. This exposes the differing degrees of liminality they are experiencing and the uncertainty that effects and shapes their journey, describing their *between states (part A)*. Students are able to navigate their resistance to the structural bounds of their inhabited community and develop their narratological approach to sharing the stories of their lives to discover and land in safe associative spaces. Digital storytelling provides students with a sense of agency over both what they share, and how they represent their experiences as markers for a better education at their university, in similar ways to how they use social media for identity making in their work, life and play (Timmis & Muñoz-Chereau, 2022).

The SEDS model has students and graduates acting as research leads, conversants, data analysts and participants, which enables the opportunity for *co-design (part B).* Co-design de-privileges (although not entirely) the authority and power of the institution and triggers opportunities for dialogue, listening and authentic collaboration. The perceived need to be anonymous over fear of reprisals in online surveys (Buchanan, 2020) is not part of the SEDS approach. This model is predicated on the ownership of identity and representation of authentic and uncertain experiences, in part because it replicates many of the co-design tropes of social media (Hausknecht et al., 2019) including identity formation and assertion, creativity and the capacity for collective engagement leading to the desired outcome of cogenerative dialogue informing curriculum transformation.

The digital stories are shared with other students in the cohort as both single stories and edited narratives intercut between participants, taking on a recurring and lasting life of their own. The capacity for anyone who watches these stories to sense themselves and their own experienced or yet to be experienced lives, either explicitly through knowing these students by face or name, or tacitly by empathising with their experiences, is critical to how they create a supportive and fertile rationale for the ways in which pedagogical change and connected curriculum are both designed and received by students (*association – part C).* It builds a sense of community within the project, centred on trust and collective understanding of the experiences that students are having. As these digital stories are shared widely on social media and act as influencers of action and behaviour, ownership and representation by the community are critical to ensuring authenticity and relevance to the cohort.

### A. Between states—Design

Students transitioning into higher education exist in betwixt states, where students feel they are in-between spaces without identifying or rightfully ‘being’ legitimate members of the university community (Palmer et al., 2009). As higher education becomes increasingly transactional and driven by employability agendas, within their curricular experiences students can feel between the states of being a learner and a professional (Grady et al., 2014). The SEDS model supports the sharing of stories of transition into and through liminality, exposing deficits of sociality and contradictions in professional and personal identity and behaviour of students.

In both case studies, the stories collected represented journeys through betwixt states. Students shared how they leveraged technology and social media presences for life and learning, making connections inside and outside the campus. Some of their experiences were rent with the uncertainty as to whether their behaviours were what was ‘expected’ of a student, as this student noted:

*You talk to people in class and stuff, and that's all right. But in between classes, especially if you have long breaks, you don't really know who to talk to. You don't really know where to go. I remember my first semester, just wandering around, not really knowing what to do.* WLPL (2018)

The SEDS model interprets the student experience as organic and uncertain, as opposed to summative and terminal in nature engendering it with a sense of dynamism and fluidity, assuming the insights revealed were not explicitly retrospective exploring the uncertainties and ambitions of a future yet to occur, nor solely their own. This data provided the projects with a very clear lens to understand the behavioural relationships between curricular action and how it is received by a student population immersed in a wider construct than the classroom, as this student from the LSE noted:

*There's the debate of whether technology closes or increases distances between people. I agree there are great benefits from technology, and I will always support technological advances. In a way though, I'm worried that technology makes everyone so accessible to each other, which makes things a lot less private between people. People are also more concerned about online presence for social and career reasons*. LSE2020 (2017)

The first step of the design stage of the SEDS model is to admit there are gaps in knowing, either within the student cohort or the institution itself. The importance of recognising this state of *not knowing* is an emancipatory one as student satisfaction or happiness are not absolute notions. They are rent with stress, freedoms, and experienced as behavioural uncertainty through which students interpret the motivations and actions of the university. The second step is to determine objectives and a focus for the project. The SEDS model uses the constructivist notion of understanding a phenomenon as it emerges through qualitative data to provide an intimate insight into the student experience without isolating it from the social setting within which it occurs (Charmaz, 2009). Critical lenses or foci enable the capability to understand the student experience in ways that support the co-design of curriculum in non-deterministic ways. It also allows for a focus to become a framing tool exploring more complex interrelationships as they influence curricular change.

In the WLPL project, the objective was to understand the ways in which the student cohort experienced their business education and how they chose to prioritise their lifeload and their learning load (Hews et al., 2022). The focus used by the project varied from understanding the importance of connection (2019) through to the meaning of flexibility (2022). Centring the conversations on these foci exposed the intersectionality that defined their student experience, as well exploring the complex jigsaw of priorities and interpretations of the educational actions they experienced.

*I'm (not) like, oh, hey, I added X amount of people on LinkedIn or made X amount of friends. It was more just a different way of viewing where I can see myself long term in both my professional career and personal life as well. Because sometimes at uni, you're kind of stuck in your own little bubble. *WLPL (2018)

The third step of the design stage is to define a thematic frame, which sets the tone for conversations and stories. The use of thematic frames strips the methodology of the rigidity and formality of online student satisfaction surveys and influences how the interpersonal interactions and relationships between the actors and students shape the degree to which students are willing to expose their feelings, emotions, and reflections on camera. A thematic frame constructs the ground rules of the relationships and trust between the project team and the students and provides the guiding rails for the objectives and the focus.

LSE2020 utilised the thematic frame of *casual conversation*. By eschewing the tropes of interviewing such as structured question and answer formats the project team ensured the students were represented as real people, living real lives, and that they had agency over how their experiences fed into the co-design process. It was a chat between peers and not an interview, which altered not just the dynamics but the willingness to share vulnerability, strength, humour, and fear which significantly enhanced the authenticity of the stories.

*I think I use social media to express my opinions sometimes, I try to be careful because I know I am very exposed at the same time and those things can be used against me…you might sometimes not feel like what you’re saying is super important, but someone might read it and identify or find it really useful for other things, so yeah, I think the more discussion and debate about things the better.* LSE2020 (2017)

### B. Co-design—Delivery and analysis

The co-design of knowledge by students and teachers is a social process, and one where new forms of knowing emerge both from the creation and the social validation of that knowing by other stakeholders (Kangas, 2010). The next two stages of the SEDS model embed co-design principles in the methodology to ensure that authenticity and ownership are central to the data that supports both the dialogue and the co-design of curriculum. Designing who participates in the storytelling process and how the stories are collected and analysed is critical to support the ways in which the model can elicit the required emotional and intellectual investment from participants. In both projects, a recent graduate led the team of current or former students acting as conversants. The project team acted as insiders rather than outside observers of the social phenomena of learning. In the earlier projects this was not by design, rather the expediency of available staff, but as the projects evolved it became critical to the design, avoiding power imbalances or the perception academics were invading student privacy (Weller, 2011).

Individuals or groups of students are engaged without pre-determined screening or sampling criteria, other than being students at the university. Approaching students in situ and engaging them on campus or online, collecting stories and filming them in the places where they study, converse, and work ensures the situational comfort and sense of agency for the participants. The use of digital media to record the stories changes the dynamics of the conversation, with students engaging performatively, and with identifiable agency of who they chose to be and represent, knowing that what they say would be shared with others and was not anonymous. Conversations are not structured but evolve in naturalistic ways. The use of an agile media approach facilitated by handheld cameras and encouraging the students to hold the microphone allows for the design and evolution of different ways to engage with students, prompt discussion and generate insights with reliability of the data not emerging from the replicability of the methodology but from the authenticity of the student voice being shared *in the moment*.

The team in LSE2020 started each conversation with an abstract question such as ‘*what will learning technology look like in 2020?*’. The aim was to prompt the sharing of current experiences through the frame of hypothetical *yet to be experienced* future states. The result was stories defined by narrative structures spanning past, present and future experiences, offering deep insights unfettered by summative criteria reflecting on the experienced.

*In the future I would like to see a closer social media connection between students and teachers. I think it will create a more intimate link between us.* LSE2020 (2016)

The principles of co-design inform the analysis stage of the model. The reflections of the project teams interpreting the stories offer an inductive lens to understand the experiences being shared. Data analysis is not limited to the team but can also include the participants themselves. The collective analysis of stories helps researchers to recognise how their own views, perspectives and background influence how they engage with the gaps in knowing (Mackenzie & Knipe, 2006). The use of inductive reasoning to interpret stories of uncertainty seeks to find patterns, rituals or resemblances in the experience of students where there is no theory or common understanding to test (inherent in most student experience surveys) (Gioia et al., 2013).

The analysis stage can draw on different qualitative methodologies. Both institutions used modified forms of constructivist grounded theory to inform the collective analysis of the stories which were transcribed and broken down into thematic storyboards, which exposed the stories through connecting different themes and patterns. Major themes were then identified through a guided discussion with quotes and reflective anecdotes exploring the theme reassembled on large posters to form connected or dichotomous narratives that formed the summarised stories that were shared back to the curriculum co-design teams. This analytical methodology enabled social-media like discursive discussions, extending past the reflections ‘in the room’ and into emails, the iterative writing of the report and the collective editing of short-form videos.

### C. Association—Sharing

The fourth stage of the SEDS model is to determine how the stories and the analysis are shared amongst the community. The sharing of stories closes the loop of feedback, exposing them to peers, future students, and staff. It shares experiential reflections on transition between students who may never have had the chance to connect. The stories can help current or future students to understand their own gaps in knowing or their reactions to being betwixt or feeling uncanny. The sharing of stories ameliorates the sense of abstraction that anonymous feedback can create. The faces, voices and identities of the students are visible, lasting and can resonate past their initial connection and analysis. The sharing of student stories is a form of rhizomatic social pedagogy, where connectivity and active modes of engagement and learning were spawned from the telling of asynchronous and sometimes disconnected stories (Benmayor, 2008; Stewart, 2017). The stories are in effect a constructed sociality, one that can be shared and consumed surreptitiously through screens on campus or directly by watching them on social media. Rhizomatic conversations then spawn organically between students who have only engaged with the stories asynchronously without knowing the students telling them. The stories represent encounters between students both inside and outside the institution that may never have happened without the intervention of the project or the affordances of the media they are recorded on and shared through.

*Yeah, I guess meet up with new friends. Because don't be isolated. Because not only the one person is facing the problem. Maybe there are many different people are facing the same problem. But you know what? When people stay together, we'll be stronger than people who are standing alone, right? *WLPL (2018)

## How the SEDS model initiated cogenerative dialogue and influenced co-design of curriculum

### Case 1 – LSE2020

LSE2020 produced over 200 three-minute digital stories between 2016 and 2018, with the analysis of the projects published in both report and blog form (Liote & Axe, 2016; Pandolfo & Molnar, 2018; Wilson, 2016). The cogenerative dialogue that emerged from the SEDS projects was triggered by the students stories of how they used technology and social media, especially the power relationships, senses of ownership of platforms and identity and how social media represented a blurred space between work, life, play and learning activities. The digital stories exposed tensions between the technology students chose to use and platforms they were ‘forced’ to use in their studies. The students described how they deployed their own technologies (being devices and platforms that they owned or were signed-up to use like Facebook or Twitter) for study situations of their choosing, supporting metacognition, network development and sociality. When the use of these technologies intersected with their learning, complex and uncertain ecosystems of privacy, exposure, safety, and wellbeing were created that impacted directly on the deployment of technology by the institution for assessment, making and sharing. The digital stories described the students inherent awareness (although not enough to dissuade their use) of the limitations of knowledge authenticity, the primacy of voice and the validity of expertise that dominate social media interactions:

*For me, one concern would be in terms of…socialisation that they might have if we abuse the use of technology, we are probably exposed to not interact much between others, among others, so that could be a potential risk of the abuse of technology that it does facilitate a life in a lot of ways but also there is a potential risk that everybody is using a lot of technology and we forget to interact.* LSE2020 (2016)

The data from LSE2020 shifted the internal institutional discourse on the use of technology within co-designed curriculum. Each project contributed towards providing students agency over how they enacted and achieved learning through assessment by not mandating the use of specific technologies or platforms. One example of this was the deployment of students as producers projects. The co-design of assessment and skills development within these courses was significantly enhanced by steering the pedagogical co-design towards the efficacy of making and the message and away from a training course on how to use YouTube (Bryant, 2017a, b). This shift was directly informed by the digital stories told by students that questioned the intellectual rigour of YouTube (for example) without questioning their own use of it for skills reinforcement.

*…the most useful [social media] is definitely YouTube because you can find everything you need in there, you can find statistics exercises, explanations about economics concepts, you find anything you would like to find, but at the same time it’s also fundamental to have the proper resources, for example, the LSE Moodle which is an invaluable resource for a student.* LSE2020 (2016)

### Case 2—WLPL

The aim of the WLPL project was to represent the authentic experiences of student learning and locate those experiences within the fuzzy boundaries of what it meant to be a student studying at the University of Sydney Business School (Yu & Bryant, 2019). Between 2018 and 2022, around 320 students have participated in the project across four iterations, producing over sixty-five hours of stories. The WLPL projects extended the duration of the stories from the shorter duration (5–10 min) in LSE2020 to a longer form, sometimes running up to 30 min. The extended conversations were enabled by flash cards and mind-mapping applications on iPads acting as narrative anchors for more exploratory storytelling. For example, students were shown the word *connection* (which was the focus for the study) prompting stories of the importance of being together or the impacts of being alone:

*You talk to people in class and stuff, and that’s all right. But in between classes, especially if you have long breaks, you don’t really know who to talk to. You don’t really know where to go. I remember my first semester, just wandering around, not really knowing what to do.*

WLPL (2018)

The stories revealed varying states of transition, development, growth, and reflection often starkly (and critically) described. The analysis identified several transient and uncertain states, represented as dichotomous perspectives on social engagement and interaction, teaching and learning, work, career, and their identity as people and professionals. These states of transition affected how students interacted with other students, academics, and their discipline, but also reflected their liminal journeys towards certainty. The demonstrations of uncertainty deprivileged the assumptions made by the Business School of a clear and well-defined pathway towards employment. This changed how the Business School designed assessment to simulate authentic approaches to work, incorporating a focus on the critical global and local challenges that were concerning students, enabling greater opportunities for formative assessment and feedforward and building in student agency over the voices, perspectives and cases they wanted to engage with during lectures (Bryant, 2022).

*I think, personally, I want to go and see the world first. And with my degree I feel like I don't want to go through a straight-line career path through business, move up the ladder. I want to do something creative, maybe join a start-up business, maybe do something along those lines.* WLPL (2018)

## Discussion and conclusion

The objectives of the SEDS model are essentially pedagogical, supporting teachers, developers, and educational managers to incorporate the authentic student experience in the co-design of teaching and learning practices through asynchronous cogenerative dialogue. Digital storytelling is a platform for acquiring a deeper, more personal, and complex understanding of the experiences of students as they traverse their education and reside within and between uncertain spaces. The technology used to create, share, and then analyse these digital stories in part defines the outcomes of the projects, determining how identity, connection and technology can shape learning and practice. Embedded into the co-design of teaching and learning, digital stories expose demonstrations of *not knowing* and locate the lived experiences of uncertainty into how a university structures and designs the student experience.

The stories in both LSE2020 and WLPL represented the positionality of students within sequences of liminal space (personal, professional, cultural, technological, and educational) that intersected with their shared expectations and outcomes of studying at university. For some of the students, residing and learning within these liminal spaces was disruptive and uncomfortable, exposing the unsettlement of the student experience, the understanding of which is critical to the effective co-design of diverse and supportive learning. The digital stories described how students chose to articulate and alleviate the traumas of their transition, by building and maintaining connections, networks, and friends, learning how to break and adjust those connections when necessary, and where to advocate for and use technology and social media:

*Perhaps, sometimes students don't really want to talk about study. What about they just want to talk about career decisions or just lifestyle? But more or less, what if I just want to hear your opinion on certain less academic product, like more life stuff.* WLPL (2018)

The SEDS model does not replace the need to engage in a varied portfolio of student experience data collection models deployed across the sector. Many of these methodologies have controllability, granularity and metrics that can disaggregate the student experience to a unit, cohort or program level, albeit through a consumerist lens (Sharpe, 2019). They can also be deployed at scale, which is difficult in the SEDS model. The data that comes from deploying the SEDS model represents an entirely different set of perspectives that methods like online student satisfaction surveys are able to articulate. The capability for a rhizomatic engagement with the student community, not just by academics and developers, but by past, present and future students enables the co-design of more authentic assessment and embedded career development opportunities and interventions. The capacity for a single digital story to be seen by thousands of other students and act as a catalyst for association or dissonance is an incredibly powerful and effective antecedent for co-design and cogenerative dialogue. It is also an organic representation of the complexity of the student cohort, which can grow throughout the academic year instead of emerging summatively after the students have graduated. The SEDS model creates lasting objects that resonate past their immediate collection and benefit from the repeated sharing and co-creation on social media to a progressively larger community inhabiting similar spaces as some or even one of the students, offering designers longitudinal data and lasting markers of the student journey. The digital stories challenge the perceptions or realities of a consumerist, transactional nature to curriculum design. It exposes the betwixt states of students to designers, academics, and most critically other students who, through the stories, may no longer feel alone or isolated in their experiences of higher education and can choose to engage in co-design or participation in radically different ways knowing they are connected to students who shared their experiences through digital storytelling. It provides both students and staff participating in a co-design process with rich senses of the reality of the student experience, as it is happening, and in a way that more directly and resonantly than other forms of students data, informs the designers understanding of the yet to be experienced, which itself whilst still a future that it yet to be written, is populated by the future versions of the students and their successive next cohorts, the future members of the university community seeking their own forms of happiness and satisfaction as they embark on the uncertain journey through liminality.

## Data Availability

All the data in this article has been published in cited works or is available on YouTube in video form.
